# Patients’ Preference to Bedside Teaching Encounters in Four Major Wards in a Tertiary Care Centre

**DOI:** 10.31729/jnma.4775

**Published:** 2019-12-31

**Authors:** Calvin Ghimire, Sajan Acharya, Abhiskar Thapa, Asha Shrestha, Deep Basnet, Kripa Rajak

**Affiliations:** 1 Patan Academy of Health Sciences, Lagankhel, Lalitpur, Nepal

**Keywords:** *acceptability*, *communication*, *medical students*

## Abstract

**Introduction:**

Patient interaction is a vital aspect of medical education. Bedside teaching encounters involve clinicians, medical students, and patients, and comprise a formative and focused activity. Patients’ willingness to cooperate and contribute to the education and training of medical students provide better teaching opportunities. The study aims to find the patients’ preference to bedside teaching encounters in four major wards in a tertiary care center in Nepal.

**Methods:**

This descriptive cross-sectional study was performed in four major wards in a tertary care centre from June 3, 2015 to July 3, 2015 after receiving ethical approval. Convenient sampling was done. Data was collected in Microsoft Excel and analyzed in Statistical Package for Social Sciences 13.0. Point estimate at 95% Confidence Interval was calculated along with frequency and proportion for binary data. Subgroup analysis was done on the basis of demographic variables.

**Results:**

Seventy-eight (77.2%) patients preferred bedside teaching encounters among 101 participants (77.12-77.28%) at 95% Confidence Interval. Among which, females, age ranging from 16 to 32 years, education below secondary school and with hospital stay < 4 days were most common.

**Conclusions:**

The results showed that most of the patients preferred bedside teaching encounters which was congruent with the other national and international studies.

## INTRODUCTION

Interactions with patients are central to medical student education. Bedside teaching encounters (BTE) involve clinicians, medical students, and patients, and comprise a formative and focused activity through which students learn both the “what's” and “how's” of physicians.^1^ It can promote contextual and clinical learning, improve communication and professional skills and initiate the development of future doctor-patient relationships.^2,3^

Studies have shown that the likelihood of patients agreeing to be involved in medical education depends on the patient, the student and the procedure being undertaken. Patients’ willingness to cooperate and contribute to the education and training of medical students provide better teaching opportunities.^2,3^

The study aims to find the patients’ preference to bedside teaching encounters in four major wards in a tertiary care center in Nepal.

## METHODS

A descriptive cross-sectional study was conducted among 101 patients in Patan Hospital, Lalitpur, Nepal after obtaining ethical clearance. The study was conducted from 3^rd^ June to 3^rd^ July 2015. Patients with age>16 years from four major wards; surgery, medicine, orthopedics and gynecology/maternity were included in the study. Patients who were below 16 years of age, who did not give consent, and who were mentally challenged, under psychiatric consultation and antipsychotic medications were excluded.

Sample size was calculated using the following formula,

$$\begin{array}{ll} \text{n} & ={\text{Z}}^{\text{2}}\times \text{(p}\times \text{q)}/{\text{e}}^{\text{2}}\\ & ={(\text{1.96)}}^{\text{2}}\times (\text{0.5}\times \text{0.5)}/\text{0}{\text{.1}}^{\text{2}}\\ & =\text{(3.84)}\times \text{(0.25)}/\text{(0.01)}\\ & =96.04 \end{array}$$

Where,

n= sample size
p= prevalence, 50%
q= 1-p
e= margin of error, 10%
Z= 1.96 at 95% CI

Sample size calculated was 97. Taking non-response rate of 4%, the final sample size was 101. Convenient sampling was done.

A pre-validated questionnaire was adopted from a study organized in teaching hospitals of the Faculty of Medicine of Kuwait University.^4^

Data was collected and entered in Microsoft Excel and analyzed in Statistical Package for Social Sciences version 13.0. Point estimate at 95% Confidence Interval was calculated and frequency and proportion for binary data. Subgroup analysis was done on the basis of age, gender, days of hospital admission.

## RESULTS

Seventy-eight (77.2%) patients preferred BTE among 101 participants (77.12-77.28% at 95% CI). Similarly, 23 (22.7%) patients did not prefer BTE. Fifty-five (80%) females preferred BTE than males among those who preferred BTE. Similarly, those with age group 16-32 years, education of below secondary and stayed in hospital for<4 days preferred BTE (Table 1).

**Table 1:** Demographic characteristics of participants who preferred BTE.

Demographic variables	Division	n (%)
Gender	Male	23 (20)
	Female	55 (80)
Age	16-32 years	45 (57.6)
	32-84 years	33 (42.3)
Education	Illiterate	14 (17.9)
	Below Secondary	30 (38.46)
	Below Higher Secondary	15 (19.23)
	Below Bachelors	15 (19.23)
	Masters and above	4 (5.12)
Hospital Stay	>4 days	28 (35.89)
	<4 days	50 (64.1)

**Figure 1:**
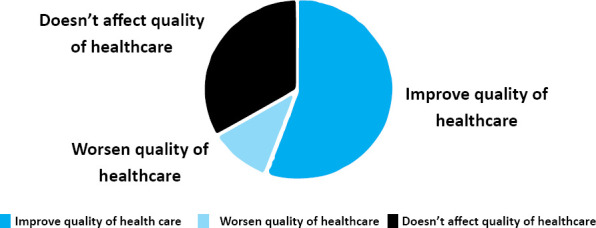
Effect of the presence of medical students in quality of health care.

Fifty-six (55.4%) patients mentioned that it would improve the quality of healthcare, 35 (33.7%) mentioned it would have no effect on the quality of healthcare provided and 10 (9.9%) mentioned it would worsen the quality of health care (Figure 1).

## DISCUSSION

Seventy-eight (77.2%) patients preferred BTE among 101 participants. The results in our study were congruent with other national and international studies.^5-7^ Many larger studies do not suggest that a healthcare facility's teaching status on its own markedly improves or worsens patient outcomes.^8,9^ In a similar study organized in teaching hospitals of the faculty of medicine of Kuwait university conducted in 995 patients in 14 teaching hospitals higher acceptance of students by patients was found. Echoing our study majority of the patients in their study, 436 (46.8%) patients believed that the presence of medical students in hospitals improves the quality of health care.^1^ Studies from different parts of the world imply positive connotations to the presence of medical students in the ward.^9-12^ Frequent and larger studies in this domain can assist medical schools in designing their curriculum and help patients understand the importance of training medical students. It also helps develop an institutional culture of gratitude towards patients who really are the most important part of medical education.^13,14^ Many studies conclude that even unprepared patients see themselves as contributors to teaching, and their capacity in this respect is probably under-utilized.^12^

The limitation of our study is as this study was conducted in smaller settings so the results cannot be generalized. It is highly recommended for the study to be done in larger settings.

## CONCLUSIONS

The result showed that most of the patients preferred bedside teaching encounters which was congruent with the other national and international studies.
